# Application of project-based service-learning courses in medical education: trials of curriculum designs during the pandemic

**DOI:** 10.1186/s12909-023-04671-w

**Published:** 2023-09-22

**Authors:** Shih-Chieh Liao, Miau-Rong Lee, Yung-Lin Chen, Hank Szuhan Chen

**Affiliations:** 1 Department of Social Medicine, China Medical University, Taichung, Taiwan; 2 School of Medicine, China Medical University, 91, Shueh-Shih Road, Taichung, 404 Taiwan

**Keywords:** Service learning, Project-based learning method, Early clinical exposure, Pandemic, Learning motivations

## Abstract

**Background:**

Due to COVID-19, face-to-face service activities in service-learning courses have become unfeasible. To address this challenge, this study aims to integrate project-based learning into medical education’s service-learning curriculum. This study also seeks to evaluate the effectiveness of this instructional approach and identify factors that influence its success.

**Methods:**

A total of 135 first-year medical students enrolled in a mandatory 1-credit service-learning course were recruited. The course involved various service activities aligned with the needs of the local community. The students were organized into 12 groups, each working on different service-learning projects, such as raising health awareness or educating the public about specific diseases. Following the completion of the course, a questionnaire was distributed among the students to gather feedback on the course, and 122 (valid responses were collected, representing a response rate of 90.3%).

**Results:**

The results indicated that the project-based service-learning course significantly improved students’ “interpersonal communication skills,“ their ability to “learn and grow from work,“ and their sense of “professionalism” (all p ≤ 0.037). Among the various aspects of service learning, the highest agreement was observed for “executing the project,“ followed by “group discussions and project formulation,“ “overall course review,“ “review of project outcomes,“ “outcome presentations,“ “teaching proposal writing and project brainstorming,“ “sharing of service-learning experiences by teachers,“ and “sharing of service-learning experiences by teaching assistants.“ Students also found certain factors to be beneficial in enhancing the learning effectiveness of service-learning courses, including “prize money for service-learning outcomes,“ “funding for service-learning activities,“ and “consultations from medical personnel” (all p ≤ 0.01). However, “course credit” and “photography software” did not show significant effects (both p > 0.05). The most preferred resources or activities for future service-learning courses were “course credit” and “face-to-face service-learning activities.“

**Conclusions:**

The project-based learning method improved the learning effectiveness in service-learning courses. Students perceived that the number of course credits reflects the course value and plays a pivotal role in enhancing the learning effectiveness in service-learning courses. During the epidemic, students still expect to have face-to-face service activities in service-learning courses. Therefore, without the impact of the epidemic, service learning courses should return to face-to-face service activities.

## Introduction

Service learning provides medical students with opportunities for early clinical exposure [1] and improves the professional competence of medical personnel [2, 3]. During service learning, service providers gain experience and learn as they provide services that address community needs [4–6]; that is, students learn while they serve. Examples of service learning in medical education include accompanying patients who are hospitalized and their family; and encouraging youths, local residents, and young immigrants to participate in community activities [1, 7]. Service learning can not only help solve community problems but also aid students in building their medical knowledge, skills, and attitudes [8], thereby creating a win–win situation for both the community and the student. Service learning has also been reported to improve the medical ethics and competence of medical interns [3] and the eight core competencies of nurses [2].

The pandemic reduced opportunities for face-to-face contact and hampered educational activities in general [9, 10], including the implementation of service-learning courses [6, 11–14]. To overcome this obstacle and still provide students with valuable related experience, medical educators, such as those in Taiwan and Hong Kong, have used non-face-to-face service-learning activities, such as online services and phone calls focused on health education [14, 15]. Although researchers claimed that non-face-to-face service-learning courses have the same learning effectiveness as face-to-face service-learning courses [2, 3, 15], medical students at China Medical University in Taiwan have emphasized their desire for face-to-face service-learning activities in their classrooms to receive feedback and opinions. They contended that non-face-to-face service-learning activities lack direct interaction with individuals, resulting in a deficiency of immediate feedback and opinion from service recipients, which in turn leads to a lack of students’ motivation in service-learning courses. They also proposed that face-to-face services is consistent with their identity as a future medical professional [16]. In the context of the pandemic, enhancing students’ motivation towards service-learning courses has become a challenge that medical educators must confront.

Project-based learning is a student-led learning method that emphasizes learning through hands-on experiences and allows students to exert greater control over the course content, facilitating the transfer of academic knowledge and experiences to real-life applications, thereby enhancing both instructional and learning effectiveness [17–19].

In project-based learning, the design of the project is guided by the interests and needs of the students. The role of teachers is to provide advice and guidance and develop assessments tailored to students [17, 20, 21]. Based on the perspectives of project-based learning, researchers argue that the project-based learning method has eight distinct characteristics, including: (1) stimulating students’ learning motivation; (2) motivating students to identify core problems; (3) allowing students to have more power over their course by, for example, determining the nature of the project; (4) providing students with opportunities for further exploration; (5) providing opportunities for reflection and reexamination; (6) displaying the final project; (7) developing core competencies; and (8) having a curriculum that fits the teaching objectives [22].

As the name suggests, project-based service-learning courses enable service learning through projects [23, 24]. In project-based service-learning courses, students and teachers collaboratively design the project based on the learning objectives, with students assuming responsibility for project planning and execution. The teachers must guide the students during the process, assess their development, and stimulate the learning motivation of students so that the students can learn, explore, and reflect on their growth. At the end of the course, students are required to submit a final project report to describe the entire process of the service activity and engage in self-reflection during the activity, aiming to enhance overall learning effectiveness [22]. The procedures of project-based service-learning courses are as follows: determine the service to be provided according to community needs, adjust the content of the service-learning course and the skills to be developed according to the service that will be offered, engage in peer learning, engage in regular reflective thinking, establish an action plan, assess the performance of the service, and celebrate the completion of the service [24, 25]. In addition, during project-based service-learning courses, teachers are responsible for communication, negotiation, guidance, and assistance of students; for example, teachers and schools help students to contact the institutions where students would serve [25].

To improve the learning motivation of students in service-learning courses, we implemented a project-based service-learning course in the medical school at China Medical University in Taiwan. This study aims to elucidate the ideal design of project-based learning courses as perceived by students, which can serve as a foundation for future developments in medical education-related course designs.

The following three research questions guided this investigation:

Research Question 1: Can a project-based service-learning course improve students’ learning effectiveness?

Research Question 2: Can the learning resources provided by schools improve students’ learning effectiveness in a project-based service-learning course?

Research Question 3: What learning resources do students most desire in project-based service-learning courses?

## Research method

### Curriculum design

The service-learning course in this study was a compulsory 1-credit course in the first semester for first-year medical students. The service-learning activities adopted in this course were aimed to address the needs of the neighboring community. A total of 135 first-year medical students were divided into 12 groups, with 10–12 students per group. The following service activities were adopted: introducing environmental issues to the public (two groups); introducing particular diseases to the public (three groups); teaching junior high school and elementary school students healthy diet knowledge (two groups); improving the sports health knowledge of kindergarten students, elementary school students, university students, and the public (four groups); and sharing experiences related to service learning with high school students (one group). Each group planned and executed the service-learning project, and a teacher and a second-year medical student, serving as a teaching assistant (TA), guided the project and shared their experience related to service learning. Due to the COVID-19 pandemic in Taiwan, online service-learning activities were the only option available to students.

**Fig. 1:**
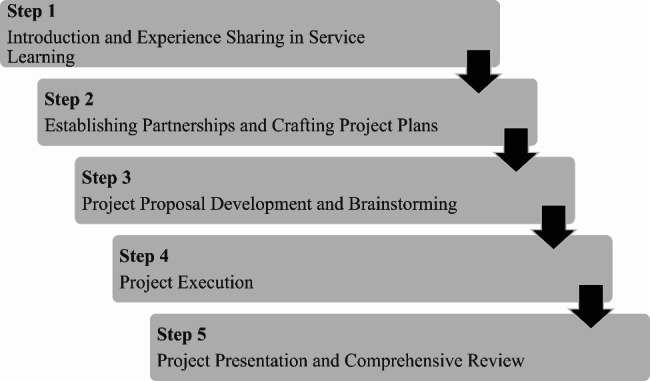
Service-Learning Journey: From introduction to comprehensive evaluation

Drawing upon insights derived from previous research on project-based learning [24, 25], we meticulously structured the course into a cohesive sequence encompassing five interconnected procedural steps. Step 1, Introduction and Experience Sharing in Service Learning, involved providing students with an introduction to service learning, during which teachers and TAs shared their own experiences relevant to service learning. In Step 2, Establishing Partnerships and Crafting Project Plans, each student group established partnerships with institutions they intended to serve and crafted their projects by carefully considering the specific needs of these institutions. Step 3, Project Proposal Development and Brainstorming, involved instructing students on how to draft project proposals and encouraging them to brainstorm ideas for their respective projects. Subsequently, each group engaged in discussions and finalized their project plans. Step 4, Project Execution, encompassed the actual implementation of these projects by the students. Step 5, Project Presentation and Comprehensive Review, entailed students presenting the outcomes of their projects, which were then assessed by both students, teachers, and TAs. Furthermore, students, teachers, and TAs conducted a comprehensive review of the entire course and celebrated the successful completion of the entire endeavor (Fig. 1). Drawing upon previous literature recommendations for project-based learning course design [17, 20, 21], throughout the entirety of these steps, teachers and TAs engaged proactively in dialogues with students concerning their projects, while also facilitating consistent evaluations and contemplations on the service-oriented facets.

### Questionnaire

The questionnaire comprised three distinct sections. The first section explored the effectiveness of the service-learning course for both service recipients and providers (open-ended questions). The second section examined the relationship between the procedures and resources of the service-learning course and learning effectiveness (19 questions rated on a 5-point Likert scale, with 1 indicating “strongly disagree” and 5 indicating “strongly agree.“ We reported the proportion of students who responded “strongly agree” and “agree” for each question). The third section investigated students’ anticipated learning resources for future service learning courses (open-ended questions).

### Participants

The study comprised 135 first-year medical students who were enrolled at China Medical University, Taiwan, and participated in a service-learning course during the Fall semester of 2021. In January 2022, following the course, we distributed questionnaires to all the students and received 122 valid responses, representing a response rate of 90.3%. Due to the brevity of student responses in the open-ended questions and the inability to identify the respondents, in February 2022, we randomly selected 12 students to assist in validating the responses to the open-ended questions.

### Research ethic and Rights of participants and informed consent

This study was reviewed and approved by the Institutional Review Board of China Medical University (IRB: CRREC-112-035). All methods were conducted in accordance with the relevant guidelines and regulations.

Before administering the questionnaire, the researchers explained to the students their rights and the confidentiality of their personal information. For example, the questionnaire responses were anonymous, and the researchers could not trace the students. Furthermore, the data acquired were for research purposes only, and the students were free to withdraw from this study at any time. Written informed consent was obtained from all the students.

## Results

### Effect of service-learning activities on service recipients

In total, 115 students (94.3%) believed that their service learning helped their service recipients. However, seven students (5.7%) expressed the belief that their service activities did not sufficiently benefit the service recipients due to limited time available and inadequate support provided. Students commented that the focus of service learning activities should not only be on imparting knowledge to the service recipients but also on assisting them in behavior change. Therefore, students perceived that the impact of service learning on the service recipients was insufficient. The results are presented in Table 1.

**Table 1:** Evaluating the effectiveness of service-learning from the perspective of students engaged in the service activities

Did service learning help the service recipient?		
	Number of responses	Example
Sufficient	115	(1) Maybe not as helpful as we think, but people should be able to approach children with developmental delays in a more friendly manner. (2) Yes, but I think the effect was limited due to the limited time. (3) Yes, we passed our experiences to high school students, and we must have provided them with a different perspective on learning. (4) The public understood more about students with special educational needs. (5) We raised the awareness of many junior high school students about face masks and pandemic prevention and the threat used face masks posed to the environment. (6) I think it was helpful but inefficient.
Insufficient	7	(1) I do not think it (the effectiveness of the service activity) was sufficient due to the limited time. (2) I think we raised awareness about the importance of proper diet for health, but it cannot make the right eating behavior change every day
Did service learning cause inconveniences to the service recipient?		
No	104	
Yes	18	(1) The course content we provided was too easy, and they knew some of it already. (2) Earlier posts were flooded by later posts. (3) We have never actually contacted the core service recipients because we do not understand this field. (4) This semester, we used online services. Therefore, the service recipients had to accommodate us, which might inconvenience them.

A total of 104 students (85.2%) believed that the service-learning activities did not cause inconvenience for the service recipients, with 18 (14.8%) students having the opposite opinion. Because the service learning was conducted online, the service recipients had to cooperate with the service providers.

### Relationship between the procedures and resources of service learning and the learning effectiveness of service learning

The Cronbach’s alpha value of the whole questionnaire (19 items) was 0.93. The questions were divided into three dimensions, namely the growth of students from service learning (six items, Cronbach’s alpha = 0.87), the relationship between the procedures and learning effectiveness of service learning (eight items, Cronbach’s alpha = 0.93), and the relationship between the learning resources and learning effectiveness of service learning (five items, Cronbach’s alpha = 0.79); the results are presented in Table 2.

**Table 2:** The outcomes of service learning for students and influencing factors such as procedures and resources (19 items, Cronbach’s alpha = 0.93)

Item	Proportion of students who strongly agreed or agreed (%)	Ranking	p-value
Growth of students from service learning (6 items, Cronbach’s alpha = 0.87)			
Interpersonal communication skills	94.3	1	< 0.001
Ability to learn and grow from work	90.2	2	< 0.001
Professionalism	59.8	3	0.037
Medical knowledge	56.6	4	0.174
System-based practice	45.9	5	0.415
Patient care	43.4	6	0.174
The procedures of service learning and the learning effectiveness (8 items, Cronbach’s alpha = 0.93)			
Executing the project	91.8	1	< 0.001
Each group discussing and formulating their project	89.3	2	< 0.001
Review of the outcome	83.6	3	< 0.001
Overall review of the course	83.6	3	< 0.001
Presenting the outcome	82.8	5	< 0.001
Teaching students how to write the proposal and brainstorm for the project	82.0	6	< 0.001
Sharing the experiences of service learning of teachers	74.6	7	< 0.001
Sharing the experiences of service learning of TA	73.6	8	< 0.001
The relationship between learning resources and learning effectiveness of service learning (5 items, Cronbach’s alpha = 0.79)			
Prize money for service-learning outcome	76.2	1	< 0.001
Funding for service-learning activities	69.7	2	< 0.001
Consultations from medical personnel	65.6	3	0.001
Course credit	54.9	4	0.319
Photography software	51.6	5	0.786

### Growth of students in service learning

Among the items related to student growth, the highest proportion of agree or strongly agree ratings was observed for “interpersonal communication skills” (94.3%), followed by “ability to learn and grow from work” (90.2%), “professionalism” (59.8%), “medical knowledge” (56.6%), “system-based practice” (45.9%), and “patient care” (43.4%). In service learning to improve their “interpersonal communication skills”, “ability to learn and grow from work” or “professionalism”, the chi-square test results showed that the proportion of students who answer strongly agree or agree is significantly higher than the proportion of students who are neutral, disagree or strongly disagree (all p ≤ 0.037; Table 2).

### Relationship between the procedures and the learning effectiveness of service learning

Among the items related to the procedures and learning effectiveness of service learning, the highest proportion of *agree* or *strongly agree* ratings was observed for “executing the project” (91.8%), followed by “each group discussing and formulating their project” (89.3%), “overall review of the course” (83.6%), “review of the outcome” (83.6%), “presenting the outcome” (82.8%), “teaching students how to write the proposal and brainstorm for the project” (82.0%), “teachers sharing their service-learning experiences” (74.6%), and “TAs sharing their service-learning experiences (73.6%). The chi-square test revealed that the proportion of students who strongly agreed or agreed that the procedures of service learning improved the learning effectiveness of service learning was significantly higher than the proportion of students who were neutral, disagreed, or strongly disagreed (all p < 0.001; Table 2).

### Relationship between the learning resources and the learning effectiveness of service learning

Among the items related to the learning resources and learning effectiveness of service learning, the highest proportion of *agree* or *strongly agree* ratings was observed for “prize money for service-learning outcomes” (76.2%), followed by “funding for service-learning activities” (69.7%), “consultations from medical personnel” (65.6%), “course credit” (54.9%), and “photography software” (51.6%). The chi-square test revealed that the proportion of students who agreed that “prize money for service-learning outcomes,” “funding for service-learning activities,” and “consultations from medical personnel” improved the learning effectiveness of service-learning courses was significantly higher than the proportion of students who were neutral, disagreed, or strongly disagreed (all p ≤ 0.001, Table 2).

### Learning resources and course design expected by the students in project-based service-learning courses

We received 108 responses regarding learning resources that students wished to have for improving the outcome of service learning (Table 3); the six resources and designs mentioned were “course credit” (n = 28), “face-to-face service-learning activities” (n = 25), “consultations from medical personnel” (n = 19), “prize money for service-learning outcomes” (n = 15), “teaching the purpose of service learning” (n = 7), and “funding for service-learning activities” (n = 2).

**Table 3:** Students’ most anticipated learning resources in future service learning courses

	Number of responses	Reflection ratio (%)
Course credit	28	24.3
Face-to-face service-learning activities	25	21.7
Consultations from medical personnel	19	16.5
Prize money for service-learning outcome	15	13.0
Teaching the purpose of service learning	7	6.1
Funding	2	1.7

## Discussion and conclusion

In the following sections, we explore our key findings, including Outcomes of the courses for service recipients, Growth of students in service-learning courses, Relationship between the procedures and learning effectiveness of service-learning courses, and Relationship between learning resources and learning effectiveness.

### Outcomes of the courses for service recipients

In alignment with findings similar to previous research [2, 7, 15], this study observed that students believed that non-face-to-face service-learning activities were beneficial for the service recipients. For examples, people developed a deeper understanding about students with special educational needs; and junior high school students learned the importance of face masks for pandemic prevention and how wantonly discarded used face masks pollute the environment. However, some students contended that the service recipients benefited little from these activities.

Students commented that the focus of service learning activities are not only to provide knowledge to service recipients but also to help them change behavior. Students believed that because the activity time is too short, service-learning activities can only improve the recipient’s understanding of the importance of proper diet for health, but it cannot make the right eating habit change every day. Therefore, students did not believe that service learning is helpful to service recipients.

Eighteen students believed that non-face-to-face service-learning activities caused inconvenience to the service recipients. The students mentioned that the recipients might not have video equipment for participating in online activities. Students claimed that if the learning content might not meet the needs of recipients because some learning content might already be familiar to them or be too advanced for them, hampering understanding; in this scenario, the recipients may lose interest in the service-learning activities.

### Growth of students in service-learning courses

Our students reported that their interpersonal communication skills, ability to learn and grow from work, and professionalism improved significantly as a result of their participation in the course. In contrast to previous research indicating that service-learning activities contribute to enhancing medical ethics and core competencies among healthcare professionals [2, 3], the present study found that students did not perceive significant improvement in their medical knowledge, system-based practice, and patient care. The absence of reported improvements in the aforementioned aspects is related to the characteristics of our students.

The students in this study were first-year medical students who had just graduated from high school. They might not have understood the importance of the abilities required for clinical practice, such as medical knowledge and patient care. Thus, they provided a low rating for the learning effectiveness of their medical knowledge and patient care from service learning course was lower. Hsu et al. [1] discovered that service learning could improve the medical knowledge of first-year medical students. Their research shows that first-year medical students understand that being with patients on the ward and possessing medical knowledge increases patients’ trust in them. The results of this study and Hsu et al. [1] demonstrated that student understanding of the importance of the learning objective, e.g. medical knowledge, during service learning could improve the learning effectiveness related to the learning objective.

### Relationship between the procedures and learning effectiveness of service-learning courses

All of the eight procedures in the project-based service-learning course adopted in this study significantly improved the learning effectiveness of the service-learning course for the students. Over 82% of the students agreed or strongly agreed that the following hands-on procedures improved the learning effectiveness of the course: learning how to write a project proposal and brainstorm for the project, discussing and formulating the project, executing the project, presenting the outcome, reviewing the outcome, and reviewing the course overall. However, less than 75% of the students agreed or strongly agreed that the sharing of service-learning experience by teachers or TAs improved the learning effectiveness of the course.

The results of this study corroborated the core principles of project-based learning pedagogy, characterized by its student-centered and hands-on approach [19, 22, 25]. The students in this study indicated that they preferred hands-on procedures because such procedures provided students with opportunities for self-directed learning, self-exploration, and self-reflection, which stimulated their learning interest. These procedures also improved the learning effectiveness of the students in the service-learning course.

Unlike the student-led procedures, the sharing of service-learning experiences by teachers and TAs was a teacher-led activity. The students indicated that teachers and TAs used lectures to share their experiences related to service-learning activities in a systematic and structured manner. This approach enabled the students to understand the multiple dimensions of service learning and why service-learning activities succeeded or failed. Nevertheless, the students lacked experience in service-learning activities, and the selected service-learning projects potentially differed from the experiences of teachers and TAs. Consequently, using the experiences shared by teachers and TAs to help the students think more deeply about the design and execution of service-learning activities was challenging.

For future project-based service-learning courses, the students recommended that teachers and TAs guide students, step by step, during brainstorming and the design of service-learning projects or provide counseling instead of simply sharing their experiences in a presentation.

### Relationship between learning resources and learning effectiveness

Among the learning resources currently provided to students, prize money for service-learning outcomes, funding for service-learning activities, and consultations from medical personnel significantly improve the learning effectiveness of the course, but not course credit, and photography software. In terms of the learning resources that students wished to have in service-learning courses, course credit was mentioned most often, followed by face-to-face service-learning activities, learning resources, prize money for service-learning outcomes, an explanation of the purpose of service learning, and funding for service-learning activities. Notably, the students reasoned that receiving only one credit for the service-learning course was insufficient to improve their learning motivations and effectiveness. Course credit remained the resources most requested by students for future service-learning courses.

The students also highlighted that the low number of credits they received for the service-learning course made it challenging to comprehend the relationship between course credits and improved learning effectiveness. Because of the pandemic, the teaching method of the course was changed from the original face-to-face service-learning activities designed by the students to online service-learning activities. Although students endeavored to plan and execute the service-learning activities efficiently, they received a low reward for their time (i.e., one course credit). Consequently, the students wished to minimize the time spent on the design and execution of service-learning activities. Notably, the students argued that increasing the course credits provided for service-learning courses would make their time spent worthwhile as well as enhance their motivation to participate in service-learning courses.

In this study, both of two kinds of funding for service-related activities, prize money for service-learning outcomes and funding for service-learning activities, were significantly improved the learning effectiveness of the course; while, the students believed that prize money had a greater effect on learning effectiveness than funding for service-related activities. The financial benefit might be the reason for the above the difference of learning effects. The service-learning activities in this study were online health education campaigns and students used their smartphones and laptop computers for filming. Students did not require lots of funding. On the other hand, the prize money awarded students directly and were their nest money.

To improve learning motivation for non-face-to-face service learning courses in medical education, researchers [12, 14] argued that service-learning courses can adopt the student-led learning method for curriculum design; and, education administration can support student-led initiatives by remodeling the curriculum to include service-learning content, establishing satisfactory internal funding opportunities, and offering recognition and credit for meaningful work. We obtained similar results: the provision of course credit and funding for service-related activities improved medical students’ initiative and learning effectiveness in service learning. The students of this study were first-year medical students who had just graduated from high school and did not understand the importance of the competencies required for future professional development. Consequently, the students did not think that the opportunities for future professional development in service-learning course can improve the initiative and learning effectiveness of the service-learning course.

In accordance with a related study [15], our study revealed that a non-face-to-face service-learning course can help improve the competence of students. However, more than 20% (25, 21.7%) of students hoped to participate in face-to-face service-learning activities. They felt a consistent barrier between themselves and the service recipients during non-face-to-face service-learning activities. For example, they could not observe the recipients’ body language or receive instantaneous feedback. Even though their knowledge and competence improved, they did not connect with the service recipients directly. Some students mentioned that non-face-to-face service-learning activities were a lesson without service.

### Limitations

Our students were from a single medical school, and the project-based learning method was adopted in a single course. Therefore, our results may not sufficiently represent the concerns pertaining to the use of project-based learning in medical education.

This study incorporated five distinct service institutions, wherein the resource levels, needs, and cultural backgrounds of diverse communities impacted the types and difficulty levels of service-learning projects in which students could engage. As a result, students’ motivations and levels of engagement in service-learning may differ across these institutions, potentially influencing their learning outcomes in service-learning. Therefore, the variability in service institutions could be acknowledged as a limitation of this study.

This study faces challenges in replicability due to specific contextual factors. To address this issue and facilitate future research in this area, we have two recommendations.

Firstly, a detailed description of the integration of project-based learning into the service-learning curriculum should be provided. This should encompass instructional design, learning activities, assessment methods, and other relevant components that contribute to the incorporation of project-based learning into medical education.

Secondly, providing a comprehensive description of the service environments and the types of projects undertaken by each student group would enhance the understanding of how different service environments may influence the outcomes of the service-learning course. Including qualitative data or case studies from each service environment can offer deeper insights into the unique challenges and opportunities presented by varying contexts.

## Conclusion

Our findings suggest that the project-based learning method enhances students’ learning effectiveness in non-face-to-face service-learning courses. The stimulation of learning motivation and the provision of essential learning resources are critical factors in improving students’ learning effectiveness within project-based learning method service-learning courses. When designing service-learning courses, instructors should give due consideration to the pivotal role played by the number of course credits in enhancing learning outcomes. Simultaneously, students express a strong desire for face-to-face interactions with service recipients, as they can receive immediate feedback, which, in turn, can bolster their learning motivation. Therefore, in the absence of the impact of epidemics, service-learning courses should return to face-to-face service activities.

## Data Availability

The datasets generated and analyzed during the current study are not publicly available due to privacy concerns but are available from the corresponding author on reasonable request.
